# Dopaminergic and Cholinergic Modulation of Large Scale Networks *in silico* Using *Snudda*

**DOI:** 10.3389/fncir.2021.748989

**Published:** 2021-10-21

**Authors:** Johanna Frost Nylen, Jarl Jacob Johannes Hjorth, Sten Grillner, Jeanette Hellgren Kotaleski

**Affiliations:** ^1^Department of Neuroscience, Karolinska Institute, Stockholm, Sweden; ^2^Science for Life Laboratory, School of Electrical Engineering and Computer Science, Royal Institute of Technology, Stockholm, Sweden

**Keywords:** neuromodulation, simulation – computers, microcircuit, dopamine, acetylcholine, striatum

## Abstract

Neuromodulation is present throughout the nervous system and serves a critical role for circuit function and dynamics. The computational investigations of neuromodulation in large scale networks require supportive software platforms. *Snudda* is a software for the creation and simulation of large scale networks of detailed microcircuits consisting of multicompartmental neuron models. We have developed an extension to *Snudda* to incorporate neuromodulation in large scale simulations. The extended *Snudda* framework implements neuromodulation at the level of single cells incorporated into large-scale microcircuits. We also developed *Neuromodcell*, a software for optimizing neuromodulation in detailed multicompartmental neuron models. The software adds parameters within the models modulating the conductances of ion channels and ionotropic receptors. Bath application of neuromodulators is simulated and models which reproduce the experimentally measured effects are selected. In *Snudda*, we developed an extension to accommodate large scale simulations of neuromodulation. The simulator has two modes of simulation – denoted *replay* and *adaptive*. In the *replay* mode, transient levels of neuromodulators can be defined as a time-varying function which modulates the receptors and ion channels within the network in a cell-type specific manner. In the *adaptive* mode, spiking neuromodulatory neurons are connected via integrative modulating mechanisms to ion channels and receptors. Both modes of simulating neuromodulation allow for simultaneous modulation by several neuromodulators that can interact dynamically with each other. Here, we used the *Neuromodcell* software to simulate dopaminergic and muscarinic modulation of neurons from the striatum. We also demonstrate how to simulate different neuromodulatory states with dopamine and acetylcholine using *Snudda.* All software is freely available on Github, including tutorials on *Neuromodcell* and *Snudda-neuromodulation.*

## Introduction

The nervous system depends on fast interaction via ionotropic receptors, which are activated by a variety of neurotransmitters including glutamate, GABA, and glycine. Besides these ionotropic receptors, neuromodulation via metabotropic receptors have a profound effect on network dynamics via slower processes. On a single cell level, these neuromodulators act via a variety of receptor subtypes, which modulate the excitability of neurons and influence their synaptic properties (Nadim and Bucher, 2014). The interactions between neuromodulators and their targets are complex. For example, a single ion channel type can be modulated by several different neuromodulators (Swensen and Marder, 2000; Park and Spruston, 2012). While a single neuromodulator can affect multiple ion channel types and signaling pathways within neurons (Greengard, 2001). On a circuit level, neuromodulation of neurons and synapses can massively alter the network activity. A challenge is to bridge the levels between single neurons and the circuit to understand the role of neuromodulators in shaping network behavior (Nadim and Bucher, 2014).

There are models of both invertebrate and different types of vertebrate which attempt to bridge the gap from cellular and synaptic properties to circuit function, and where the effects of neuromodulation is considered (Lansner et al., 1998; Kozlov et al., 2001, 2009; Hamood and Marder, 2014; Nadim and Bucher, 2014; Colangelo et al., 2019). For neuromodulation, the challenge is that the effect of a single neuromodulator in a single cell setting could be very different once that cell is embedded within a circuit. In the circuit, other components, such as other neurons and synapses, would also be under neuromodulatory control and hence contribute to more complex circuit interactions (Oh et al., 2012). Therefore, computational models have to incorporate the ability to bridge these levels. Furthermore, it has been shown that different parts of a neuron (dendrites/soma) can be modulated differently depending on receptor sub-type and target (Bender et al., 2010). Such compartmental differences can be implemented in multicompartmental models where reconstructed neuronal morphologies are used and ion channel models are distributed throughout the morphology (Frost Nylén et al., 2020; Hjorth et al., 2020; Lindroos and Hellgren Kotaleski, 2021).

We use the *in silico* striatal microcircuit (Hjorth et al., 2020) as a demonstrative example, which consists of 95% striatal projection neurons (SPNs) and 5% interneurons (fast-spiking (FS), low-threshold spiking and cholinergic interneurons). Here, we simulate neuromodulation of SPNs and FS. The striatum is the input stage of the basal ganglia, a group of subcortical nuclei involved in action-selection, motor control, and habitual learning. Neuromodulation is important in the striatum and especially dopamine is essential for basal ganglia function (Gerfen and Surmeier, 2011; Surmeier et al., 2014; Da Silva et al., 2018). The cholinergic interneuron (ChIN) is a spontaneously active interneuron within the striatum, which releases acetycholine (ACh). Hence, in addition to the dopaminergic modulation, cholinergic modulation can modulate several components of the striatal network, which has extensive expression of muscarinic receptors (Abudukeyoumu et al., 2019). Furthermore, several studies have demonstrated the complex interactions between DA and ACh within the striatum, ranging from modulating dopamine release via presynaptic nicotinic receptors to direct dopaminergic modulation of ChINs via D2 receptors (Threlfell et al., 2012; Howe et al., 2019).

**FIGURE 1:**
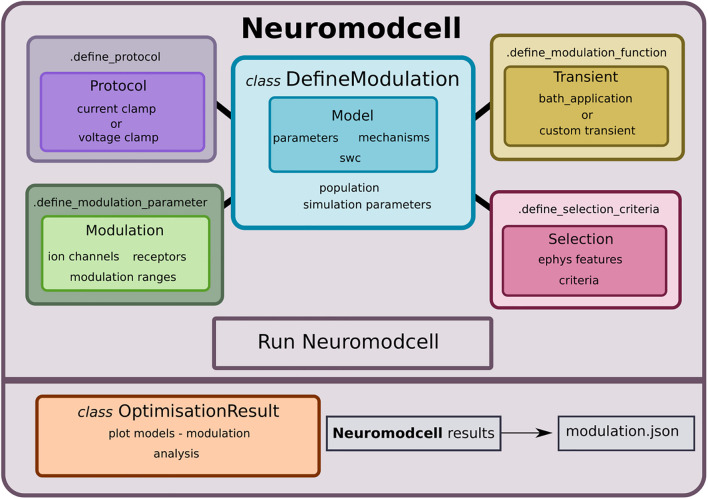
*Neuromodcell* structure with classes and methods. *Neuromodcell* specifies the model and optimization parameters using the *DefineModulation* class. The associated methods define the protocols, parameters, modulation, and selection criteria for the optimization. Following the simulation, the *OptimisationResult* class assists in loading and analyzing the results and saving the final modulation (modulation.json).

Previously, *Snudda*, a Python package for creating data-driven networks of neurons, placing synapses using touch detection between axons and dendrites and setting up large scale simulations was developed by Hjorth et al. (2021). The software included fast synaptic transmission but neuromodulation was limited to dopamine. Hence, the *Snudda* package required further development to accommodate the generalized implementation of neuromodulation, together with all the other functionalities contained within *Snudda*. Following the development of *Snudda.neuromodulation*, it was necessary to create a software for generating and selecting parameter sets which reproduce neuromodulation. Therefore, we decided to develop *Neuromodcell*, which extracts the modulatory parameters necessary to reproduce neuromodulation on a single cell level for multicompartmental neuron models (Figure 1).

In this study, we applied *Neuromodcell* and *Snudda.neuromodulation* to the dopaminergic and cholinergic modulation of the *in silico* striatal microcircuit to demonstrate the functionalities included in these software packages. In general, *Neuromodcell* and *Snudda* can be used to generate and simulate networks of multicompartmental models with a wide range of neuromodulators. Within these simulations, a variety of questions could be addressed. Hence, not merely to simulate neuromodulation, but to predict which components are important for certain features and also redundancies within the network. Furthermore, simulations of multiple neuromodulators could predict how these interact on a network level. Both *Neuromodcell* and *Snudda* are open-source and freely available on Github, for further enhancement and expansion.

## Materials and Methods

### Software Setup

*Neuromodcell* is freely available to be downloaded from its Github site^1^, written in Python with requirements specified in *requirements.txt* including NEURON^2^ and Jupyter Notebook. To install *Neuromodcell* use *pip install neuromodcell*. The software is compatible with Linux operating system and super-computer clusters (Cray XC40 system). The simulations of large networks use *Snudda* (Hjorth et al., 2020, 2021), a Python package for creating data-driven networks of neurons available from its Github site^3^. To simulate neuromodulation, within the *Snudda* framework, additional simulation classes and associated neuromodulation subpackages were created, s*nudda.neuromodulation.* To use *Snudda*, install via *pip install snudda* and follow the instructions on its wiki page (see text footnote 3).

### Multicompartmental Models

*Neuromodcell* and *Snudda* require multicompartmental models specified for NEURON simulator described by three files: a morphology file (SWC), a parameter file (JSON) and a mechanism file (JSON) including a directory with the ion channel model files (.mod files, NEURON model description language). The models used in this publication were optimized using BluePyOpt (Van Geit et al., 2016) in previous publications (Hjorth et al., 2020; Lindroos and Hellgren Kotaleski, 2021) with modification for muscarinic modulation; and include direct and indirect striatal projection neuron (dSPN and iSPN) and fast-spiking interneuron (FS) models from the striatum.

### Parameterization

To modulate the models during the simulation, we introduce additional parameters for the specific neuromodulator within the .mod files. The modulation is implemented in a phenomenological manner, where a modulation parameter is multiplied with the target (for example the conductance of an ion channel). Hence, the conductance (in this case) can be regulated through the simulation. For example, for dopamine, the three parameters are maxModDA, modDA and levelDA (using the key, *DA* to indicate dopamine), based on previous formalism developed in Hjorth et al. (2020). In general, the convention is ‘maxMod^∗^’, ‘level^∗^’ and ‘mod^∗^’ and the ^∗^ should be replaced with a specific name for the neuromodulator. The maxMod^∗^ defines the modulation degree (which can vary, with 0.6 being 40% reduction). The level^∗^ defines the transient level of modulation throughout the simulation. The mod^∗^ defines the activation of modulation (0 inactive, 1 fully active). Within the examples, dopaminergic and muscarinic modulation was included (with muscarinic modulation using key *ACh).* The list of modulated ion channels for each cell type is presented in Supplementary Table 1. For examples of .mod-files with these modulation parameters (see text footnote 1).

### Neuromodcell

*Neuromodcell* is a software for optimizing and simulating neuromodulation in detailed multicompartmental neuron models. The software loads and simulates a population of models with user-defined parameter variations of maxMod^∗^, which is how the program induces the changes associated with neuromodulation. The software also incorporates current clamp and voltage clamp parameters to simulate specific biological experiments. Hence, the user can define the simulated experiment in terms of both neuromodulator and the specific protocol to be used. Following the simulation, the parameter sets that reproduce the experimental data are selected based on a user-specified selection criteria. We provide several electrophysiological features by default, but custom features can be included.

Here, *Neuromodcell* was used to optimize dopaminergic modulation for dSPN, iSPN and FS, as well as muscarinic modulation for dSPN and iSPN. The optimizations for dSPN and iSPN were validated against data from Planert et al. (2013) and Lahiri and Bevan (2020) (see Supplementary Figure 1) for dopaminergic modulation and Lv et al. (2017) for muscarinic modulation. The dopaminergic modulation for FS was validated against data from Bracci et al. (2002).

### Neuromodulation in *Snudda*

Neuromodulation in *Snudda* is implemented as a separate simulation module called *snudda.neuromodulation*. Within the module, there are two simulation classes for neuromodulation, *SnuddaSimulateNeuromodulation* and *SnuddaSimulateNeuromodulationSynapse*. These classes represent the two strategies used to simulate neuromodulation, *replay* and *adaptive.* The *replay* mode plays a predetermined transient through the *level^∗^-*parameters and hence modulates each component of the circuit in a predefined time dependent manner. On the other hand, the *adaptive* mode originates from the fact that a neuromodulatory neuron integrated within the microcircuit could not modulate via a predetermined transient. Instead, the spiking activity of such a neuron would have to be continuously translated into an instantaneous level of neuromodulation. Therefore, an alternative approach was developed where an intermediate mechanism would integrate the spiking activity. *Adaptive* simulations couple one or several presynaptic neurons to postsynaptic neurons via an integrating mechanism called conc^∗^, which modifies the neuromodulatory parameters (*level^∗^)* in the circuit (for example concDA to levelDA for dopamine). Hence, neuromodulation within the simulation can either follow a fixed recipe (‘replay’ mode), or dynamically change based on the activity in the network (‘adaptive’ mode). The mode used within a particular simulation will therefore depend on the network structure. Using either approach, the level of neuromodulation would be updated every time step via the *level^∗^-*parameter. The *replay* mode loads an array which has set values for the *level*^∗^ for each time step of the simulation. This would suffice if the neuromodulation is synchronous and the neuromodulatory neurons are not affected by activity of the circuit. On the other hand, if the activity of the microcircuit could for example inhibit the neuromodulatory neurons, like striosomal dSPNs in the striatum, *replay* mode does not suffice and *adaptive* mode would be used instead, as the presynaptic neuromodulatory neurons can interact with the microcircuit throughout the simulation.

For *replay*, the *maxMod*^∗^, *mod*^∗^ and *level*^∗^ are introduced as *RANGE* variables. In the *adaptive* mode, which is based on pointers, the parameters are introduced with the keyword POINTERS^4^ and ‘ptr’ is added to the filename (i.e., na.mod to na_ptr.mod).

Lastly, both types of simulations require a configuration file (JSON) which is created using either *Neuromodulation* class (*snudda.neuromodulation.modulation_network*, for *replay* simulations) or *NeuromodulationSynapse* (*snudda.neuromodulation.modulation_synapse*, for *adaptive* simulations). For examples of how to use these classes (see text footnote 4).

To demonstrate the features of *Snudda.neuromodulation*, we simulated several networks using both *replay* and *adaptive* mode. Firstly, using the *Snudda* framework, a network of 10 000 neurons was created and simulated with dopamine and acetylcholine transients; and with a cortical activation at 1 s, with cortical and thalamic background activity. The network is based on previous publications (Hjorth et al., 2020) and the connection probabilities follows the diagram in Supplementary Figure 2. The transients consisted of a tonic level of ACh accompanied with a burst or pause of ACh and a DA burst. The simulations were performed on a super-computer cluster (Cray XC40 system at PDC Center for High Performance Computing, KTH). A smaller network with dSPNs was simulated to show the effect of DA and ACh without network interactions from iSPNs and FS. Furthermore, several dopamine transients with different start times were created using the *Neuromodulation* class and applied to a network of 20 dSPNs with a cortical activation lasting for 500 ms. Lastly, a network of dSPNs was simulated with presynaptic spiking neurons using the *adaptive* mode. The presynaptic neurons represented dopaminergic neurons and were simulated with and without activation (i.e., bursting). The activity of the neurons within the simulations were measured by the percentage of spiking neurons and the mean firing frequency during the cortical activations using custom Python code and Electrophysiology Analysis Toolkit^5^.

### Tutorials

A tutorial on *Neuromodcell* and an example of dopaminergic modulation of dSPN are available (see text footnote 1).

Examples of simulations using *Snudda.neuromodulation* module are available (see text footnote 4).

## Results

### Overview

**FIGURE 2:**
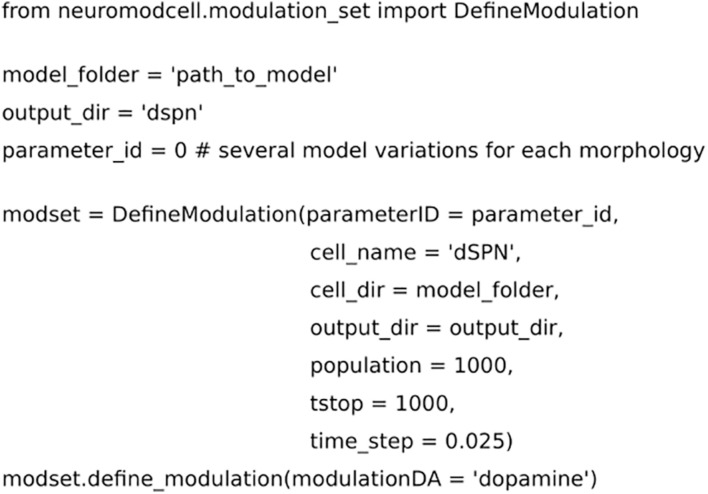
Code for setting up the parameters for neuromodulation using *Neuromodcell*. The parameter, “path_to_model,” defines the path to the model and which specific parameterID, *parameter_id*. *mod_set.define_neuromodulation*, the naming defines the neuromodulation key, DA for dopamine. The example has a simulation time of 1000 ms and population of 1000 different models.

**FIGURE 3:**
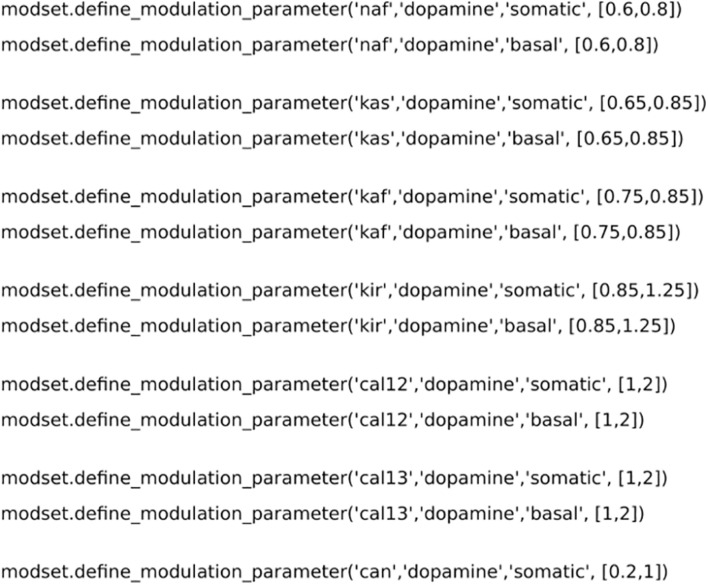
Code for setting up the parameter variation for neuromodulation. *modset.define_modulation_parameter* accepts the ion channel name, neuromodulator, part of the morphology and the intervals of modulation. Here we have used data from previous publications (Lindroos et al., 2018; Lindroos and Hellgren Kotaleski, 2021).

**FIGURE 4:**
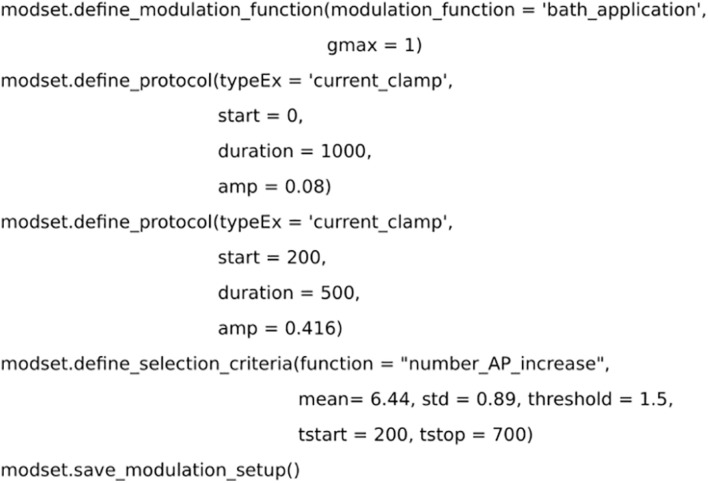
Code for setting up the selection criteria. Dopamine level is simulated as a bath application. Two simulated current clamps are added to the set up to replicate the experiment from Planert et al. (2013). The effect of dopamine is measured by the number of action potentials and compared with experimental data.

*Neuromodcell* performs parameter variation of ion channels and receptor models within multicompartmental models to simulate neuromodulation. The components of the software are presented in Figure 1 with the main class, *DefineModulation* (Figure 2) and its associated class methods, which are used to define the parameters, protocols, selection criteria and modulation for the specific cell and neuromodulator. The user specifies the range of parameter variation for each ion channel and/or receptor as well as the experimental protocol to be replicated in the simulation (Figures 3, 4). The models with parameter variations are simulated using the *optimise* module, which uses *mpi4py* for parallelization.

Following the simulation, a selection process is performed, where the models are compared to the experimental data provided in the setup (using *DefineModulation)*. The parameter variations of the models which pass validation are saved in a separate file. The code for defining the selection criteria is freely available (module *selection_criteria* and *selection_function*) and can be modified to measure a specific electrophysiological feature of the simulation, for example firing frequency or the number of action potentials.

Below, we describe the process of setting up, optimizing and selecting the models that pass validation. Following the completion of the optimization, the results are transferred to *Snudda* and simulated using *snudda.neuromodulation* module. A step-by-step tutorial is available (see text footnote 4).

### Defining Neuromodulation *in silico*

We performed optimizations of dopaminergic and muscarinic modulation for dSPN, iSPN, and FS. In the following examples, the dopaminergic optimization of dSPNs is used to demonstrate the features of *Neuromodcell*. We instantiated the *DefineModulation* class as shown in Figure 2. The class requires several arguments to define the optimization, including the model directory and the number of model variations to simulate (population). The next step is parameter variation. In Figure 3, the ion channels which are modulated by DA within dSPN (based on previous publications Hjorth et al., 2020 and Lindroos et al., 2018) were set using the *define_modulation_parameter* method within the class. The modulation can also be defined to be somatic, dendritic (basal or apical, due to the SWC naming convention) or axonal.

### Experimental Setup and Selection Criteria

The experimental data used in the example optimization was taken from Figure 6C in Planert et al. (2013) for dSPNs. The experiment used patch clamp recordings and bath application of dopamine to measure the effect of DA on dSPNs. Following the application of DA, there was an increase in the intrinsic excitability of dSPNs measured by the number of action potentials. Hence, an experimental setup was introduced into *modset*, with a current clamp protocol and bath application of dopamine (Figure 4). Furthermore, the method *define_selection_criteria* was used to introduce the measurement used in the validation step (i.e., the change in the number of action potentials). Both the *define_modulation_function* and *define_selection_criteria* can select several pre-defined functions but they can also be customized to accommodate specific transients or criteria, respectively.

### Optimization

**FIGURE 5:**
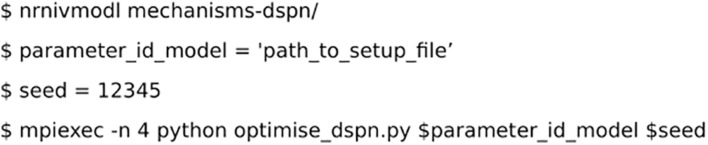
Code for running the optimization.

The optimization uses the *OptimiseModulation*, from *optimise* module in *Neuromodcell*. The optimization for dSPN used a custom Python script called optimise_dspn.py, which loaded the *path_to_model* and the specific *seed* for the optimization (Figure 5). The seed is used to randomize the modulation parameters defined using *define_modulation_parameter.* The modulation parameters sets (i.e., population) are simulated in parallel with the specific model (defined in *DefineModulation)*, as illustrated in Figure 1.

### Analysis

**FIGURE 6:**
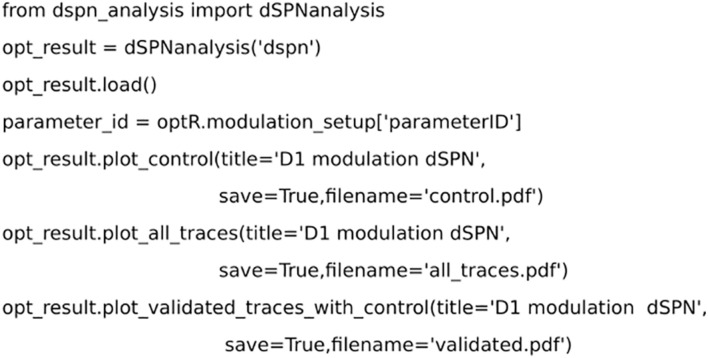
Code for analyzing the results from the optimization using *dSPNanalysis*. dSPNanalysis (*inheritance* from *OptimiseAnalysis* class) loads the results from the optimization. The results can be plotted by custom methods and the final modulation is saved.

**FIGURE 7:**
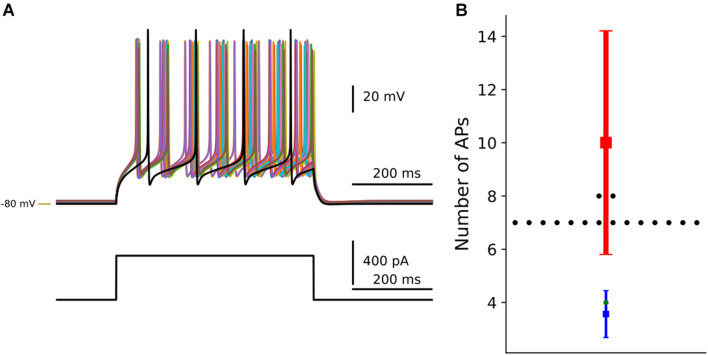
Optimization of dopaminergic modulation of dSPN. By using the *Neuromodcell* package, parameter sets which reproduce the dopaminergic modulation are applied to the multicompartmental model of dSPNs. **(A)** Simulation of current clamp recordings of dSPN. Control simulation without dopamine modulation in black and dopamine modulated simulations which passed the selection criteria (non-black traces). **(B)** The change in the number of action potentials is compared to control (in green) and the parameter sets which passed in black. The mean and standard deviation of the control behavior from Planert et al. (2013) in blue, the DA modulated mean and standard deviation in red. –80 mV marked by yellow line.

The result of the optimization was visualized using *dSPNanalysis* (inheritance from *OptimiseAnalysis* class in *Neuromodcell*) (Figure 6). The class loads the output files following the optimization and enables the user to plot and/or analyze the results. In Figure 7, the result of dopaminergic modulation of dSPN is shown with the model variations (modulated) and control simulation (in black). The dopaminergic modulation increases the intrinsic excitability of dSPNs via D1 receptors. The result of the *Neuromodcell* optimization produced model variations which reproduce the experimental data from Planert et al. (2013). The control simulation (in black, Figure 7A) has four spikes following current injection. The simulated dopamine modulation increases the number of spikes and the change in the number of action potentials are within the range extracted from the experimental data (Figure 7B).

### Transfer Files to Snudda Simulation

Following the optimization, the parameter sets which reproduce the neuromodulation are saved as modulation.json and moved to the model directory (i.e., the model is now defined by parameters.json, morphology.swc, mechanisms.json and modulation.json). The modified .mod files should also be transferred to a common *Snudda* mechanism directory.

### Dopamine

**FIGURE 8:**
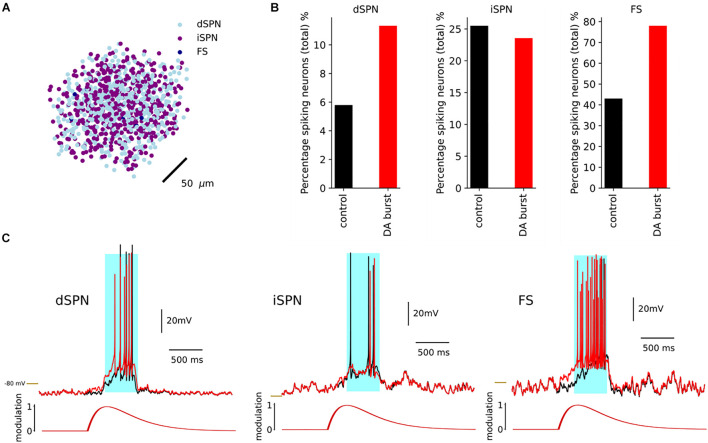
Simulation of dSPN with and without DA within a network of 10 000 neurons with 4950 dSPNs and iSPNs and 100 FS. **(A)** A network of 10,000 neurons. Here we plot the soma positions. A dopamine transients was initiated at 0.5 and a cortical stimulation at 1 s. **(B)** The response of dSPN with the dopamine modulation in red and the control in black. The dopamine modulation causes a depolarization in the dSPN which results in an increase in the percentage of spiking dSPNs. The iSPN responded with a decrease in the percentage of spiking neurons, while FS increased. **(C)** Examples of dSPN, iSPN and FS models and the response with dopamine modulation (red) and control (black), with cortical stimulation in light blue. –80 mV marked by yellow line.

Using the *replay* mode in *Snudda.neuromodulation*, we simulated a network of 10 000 (Figure 8A) multicompartmental neuron models of dSPNs, iSPNs, and FS to exemplify the effect of the dopamine modulation. The simulation included a cortical stimulation of dSPNs, iSPNs and FS at 1 s, lasting for 0.5 s. In Figure 8, the effect of dopamine on network activity is shown. Following cortical activation, 6% of dSPNs within the network were spiking. By modulating the ion channels defined in the optimization result of *Neuromodcell*, dopamine modulation increases the percentage of spiking dSPNs to the double (Figures 8B,C). iSPNs are modulated by D2 receptors, which reduces the intrinsic excitability. Hence, in Figure 8B, the percentage of spiking iSPNs within the network decreases by approximately 3%. The middle panel in Figure 8C shows an example trace of an iSPN, where the dopamine modulation reduces the number of spikes. FS are modulated by D1-like receptors, which depolarizes the membrane potential, as seen in Figure 8C. Following, an optimization using *Neuromodcell*, the parameter sets for FS dopamine modulation increase the number of spiking neurons within the network (Figure 8B). The cortical activation occurs via activating glutamate ionotropic receptor models. These receptors can also be modulated by the same formalism as previously described which leads to changes in the amplitude and release probability (Supplementary Figure 3).

### Dopamine and Acetylcholine

The neuromodulation extension to *Snudda* can also simulate multiple neuromodulators. To demonstrate this, we simulated five scenarios of dopamine and acetylcholine modulation with the same network presented in Figure 8A. As previously described, dSPNs and FS are modulated by D1 receptors while iSPNs are modulated by D2 receptors. In addition, we used *Neuromodcell* to optimize for muscarinic modulation for dSPNs and iSPNs.

**FIGURE 9:**
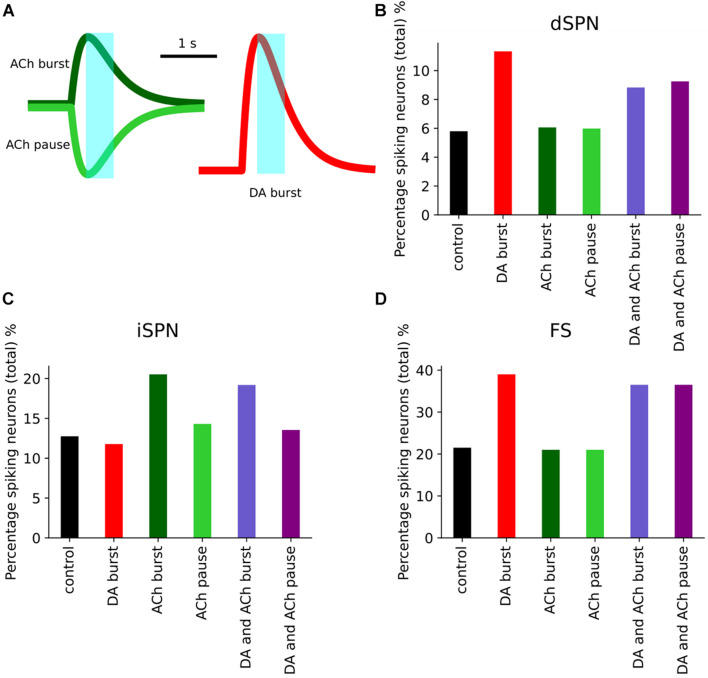
Simulation of five neuromodulation scenarios involving dopamine and acetylcholine in a network of 10,000 neurons, 4950 dSPNs and iSPNs and 100 FS. The network received a cortical simulation at 1 s, for 500 ms. **(A)** Examples of the acetylcholine (ACh) burst and pause transients and the dopamine burst transient. In light blue, the timing of the cortical activation in relation to the transients. The percentage of spiking neurons are measured during the cortical simulation. **(B)** The response of dSPNs in the five neuromodulation scenarios **(B)**, dopamine (red) produced the largest effect. The acetylcholine burst and pause (light and dark green) and combinations of DA burst and ACh burst and pause (light and dark purple, respectively). **(C)** The response of iSPNs in the five neuromodulation scenarios and **(D)** the response of FS.

In Figure 9A, we show the transients used in the simulations, which consisted of an acetylcholine burst or pause and a dopamine burst. In the striatum, acetylcholine is released continuously by cholinergic interneurons due to their spontaneous activity. Hence, the acetylcholine transients had a tonic modulation level of 0.5 with burst and pause response causing an increase or decrease (by 0.5), respectively. These transients were simulated individually but also simultaneously (DA and ACh burst; DA and ACh pause). As shown in Figure 8, dopamine increased the percentage of spiking dSPNs within the network. The acetylcholine burst also slightly increases the percentage of spiking dSPNs, although in the simultaneous simulation (Figure 9B, light purple), the effect is not additive. Instead, the dopamine burst and acetylcholine pause produces a larger response. This is contrary to previous results in Lindroos and Hellgren Kotaleski (2021), where DA and ACh had additive effects when single neurons were simulated. Hence, we performed a simulation with only dSPNs to investigate the effect of DA and ACh. Without network interactions, the effect of DA and ACh was indeed additive (Supplementary Figures 4A,C). Hence, the decrease in the percentage of spiking dSPNs could be attributed to the network interactions and the changes seen in iSPNs and FS. As described in Figure 8, the intrinsic excitability of iSPNs is decreased following dopamine modulation, while ACh modulates iSPNs via M1 receptor, which lead to the opposite effect. Compared to dSPNs, iSPNs have a larger increase in the number of spiking neurons following an ACh burst (Figure 9C). During the ACh pause, the percentage of spiking iSPNs is expected to decreased, due to the reduction in ACh levels. In contrast, the percentage of spiking iSPNs was larger than in the control. In Zucca et al. (2018), SPNs were recorded *in vivo* and cholinergic interneurons were inhibited optogenetically. They observed a reduction in the spontaneous activity of SPNs. The control simulation in Figure 9 was, however, run without any neuromodulation, which could explain the inconsistency. Hence, we simulated a small network of SPNs with a cholinergic pause, but we modified the control simulation to contain a continuous tonic level of ACh. By comparing the mean firing frequency of SPNs with and without the pause, we observed the expected reduction in SPN activity (Supplementary Figure 4). Therefore, the tonic level of ACh has an influence on the activity of SPNs. For FS, the main effect is seen within the dopamine burst modulation (Figure 9D), while for ACh pause and burst there is no visible effect (due to the lack of direct muscarinic modulation of FS).

### The Timing of Dopamine

**FIGURE 10:**
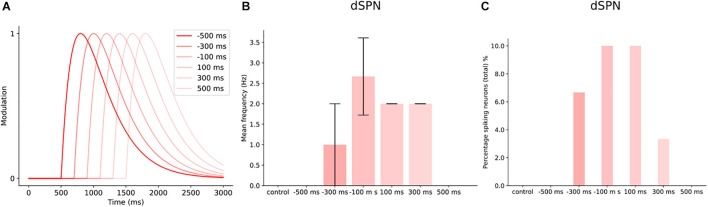
Timing of dopamine signal with cortical input in a small network of dSPNs. **(A)** Different delays in the dopamine transients with cortical input at 1000 ms, for 500 ms. **(B)** The mean frequency of dSPNs following the different DA delays. In control (without dopamine modulation), the dSPNs within the network do not spike following cortical activation. Dopamine modulation starts at 500 ms before cortical input (–500 ms), does not produce enough modulation to induce spiking. While –300 ms, increases the mean firing frequency and the following delays except 500 ms. **(C)** The percentage of spiking neurons within the network at different dopamine delays. At 300 ms, the percentage of spiking cells was 7%. –100 and 100 ms produced a similar effect on the number of spiking neurons within the network.

The user can construct different transients to investigate the effect of timing on network activity. We created a smaller network of 30 dSPNs to illustrate the effect such an investigation could have on network activity. We defined six transients which were identical, in terms of time constants and modulation level (Figure 10A). We then adjusted the start time in relation to cortical stimulation at 1s (of a 3 s simulation). The different delays caused the peak modulation to occur at different times during the cortical stimulation. Figure 10B shows that the transient −100 ms results in the largest increase in mean firing frequency. While −/+500 ms transients are not different from control. This is due to the dopamine level being too low and the peak dopamine response occurring much earlier and later compared to the cortical stimulation. The +100 ms delay produced a lower increase in the mean firing frequency but the percentage of spiking neurons were similar to the −100 ms case (Figure 10C). The user could conduct similar investigations into other features of the transients such as the time constants, tonic levels or more complex transients (Lahiri and Bevan, 2020).

### Adaptive Modulation

**FIGURE 11:**
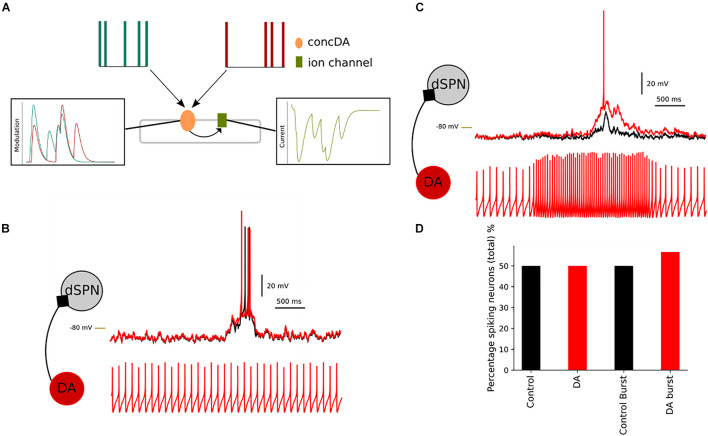
Simulating presynaptic spiking neurons and dopaminergic neuromodulation. In **(A)**, a schematic representation of the adaptive mode, concDA (orange) placed on a segment within the multicompartmental model, which receive input from presynaptic cells (red and green). Every spike input triggers a transient inside the concDA which is sent to the ion channel (green) on that particular segment, rightmost panel in **(A)** hence modulating the current. In **(B)**, a simulation of a network of dSPNs, where neuromodulation depends on presynaptic spontaneously active neurons. Compared to control (without modulation) (black), the simulation with the presynaptic neurons produces a depolarization in dSPNs (red). If the frequency of the presynaptic neuron increases **(C)**, this translates to an increase in the neuromodulation. The dSPN depolarises more than in the spontaneous case and this results in an increase in the percentage of spiking neurons. In **(D)**, the bar chart shows the comparison between control and spontaneous versus bursting presynaptic cell (and control for bursting simulation, where the connections between the cells were disabled). The percentage of spiking cells within the network increases following the dopamine modulation which occurs when the presynaptic cell firing frequency increased. –80 mV marked by yellow line.

The previous simulations have used a transient(s) to modulate the network. In Figure 11, we simulate neuromodulation using the *adaptive* mode in *Snudda*. In *adaptive* mode, the spiking activity of the presynaptic neurons are translated to modulation levels as described in Figure 11A. The top panel in Figure 11A shows the spike times of two presynaptic neurons (green and red). These neurons are connected to an integrating mechanisms called concDA (for dopamine, in orange in Figure 11A). The concDA integrates the spiking activity of the two cells into modulation levels (as seen in Figure 11A in left most box). The concDA sends the modulation level from both neurons into the ion channel model (right most box in Figure 11A). The level of current follows the modulation as the conductance of the ion channel is modulated and hence affecting the neuronal activity.

We created a small network of dSPNs to illustrate the effect of changing the firing frequency of the presynaptic neurons. We placed spontaneously active neurons to modulate the dSPNs via D1 receptors. In control, 50% of the dSPNs within the network were spiking and the spontaneous activity of the presynaptic neurons did not change the percentage (Figure 11D). Although in Figure 11B, the spontaneous activity has an effect on the membrane potential of the dSPNs. By stimulation the presynaptic spontaneous neurons, we increased the firing frequency (Figure 11C). As expected the level of neuromodulation increased as the concDA mechanism integrates the number of spikes into neuromodulation. This leads to a depolarization of the dSPN membrane potential. On a network scale, the increased firing frequency leads to simulated D1 receptor modulation and an increase in the percentage of spiking dSPNs.

## Discussion

In this article, we present a framework for simulating neuromodulation in detailed multicompartmental neuronal models being part of large-scale microcircuits, using *Neuromodcell* and *Snudda*. Our priority was to create an extension to *Snudda*, a software for large scale simulation of networks of multicompartmental models, which also simulates transients of neuromodulator(s). Firstly, we developed *Neuromodcell* which enables the user to optimize and select parameters for a specific type of neuromodulator. The neuromodulation is defined by introducing parameters within the ion channel and receptors models, hence enabling dynamic control of for example conductance. Secondly, we developed an extension to *Snudda*, which incorporates the neuromodulation parameters found using *Neuromodcell*. Thirdly, we simulated a range of different neuromodulatory states with the striatal microcircuit to show the versatility of the tool.

We simulated an example network of dSPNs, iSPNs, and FS. These neurons are modulated by dopamine D1 and D2 receptors. We used *Neuromodcell* to optimize for parameters sets which reproduced the available experimental data on dopaminergic modulation of these cell types. The simulations of dopaminergic modulation showed that DA can modulate the excitability of the network. Hence, the simulations of dopamine are in line with known effects of DA in the striatum (Gerfen and Surmeier, 2011; Maltese et al., 2021). Currently, the *Neuromodcell* optimization produces a population of modulated models, which are selected according to predefined parameters. In the future, the *Neuromodcell* optimization could be improved and utilize more elaborate optimization algorithms, for example the genetic algorithm used within *BluePyOpt* software (Van Geit et al., 2016).

Within the striatum, cholinergic interneurons release acetylcholine which modulates the network via nicotinic and muscarinic receptors. Both dSPNs and iSPNs are modulated by the M1 receptor, while only the M4 receptor modulates dSPNs (Abudukeyoumu et al., 2019). Using *Snudda.neuromodulation*, we simulated muscarinic modulation throughout the whole network. We defined a burst and pause transient with a tonic background level of muscarinic modulation to replicate the acetylcholine levels reported in the striatum. We could show that this changes the excitability of dSPNs and iSPNs which is consistent with reports (Abudukeyoumu et al., 2019). Furthermore, we could replicate the effect of ACh pause on SPNs reported by Zucca et al. (2018).

Within the striatum, there are several *in silico* investigations which can be performed using *Snudda.neuromodulation.* In Parkinson’s disease (PD), the degeneration of dopaminergic neurons results in a change in the balance between dopamine and acetylcholine (McKinley et al., 2019) and anti-cholinergic drugs were used to treat PD before DOPA therapy emerged (Carlsson, 2001). Although, the interaction between dopamine and acetylcholine is not fully understood. Therefore, future simulations could investigate how the changes of DA and ACh on the single cell level affect network activity; and potentially dissect the important components using *Neuromodcell* and *Snudda*. Furthermore, in Howe et al. (2019), they showed that cholinergic activity and DA levels were coordinated during spontaneous movement. Using *Snudda.neuromodulation* such transients could be simulated to understand how the coordination between ACh and DA affects the activity of dSPNs and iSPNs.

Recent advances in biosensor technology are enabling research to visualize neuromodulator levels within neuronal networks (Leopold et al., 2019). These biosensors can provide high spatial and temporal resolution on the action of neuromodulators like dopamine, serotonin, and opioids amongst others. Hence, a simulation platform like *Snudda*, can incorporate such data to understand how the underlying neural circuit responds to these transients.

Neuromodulation affects many aspects of neurons and neural circuits. Presently, *Neuromodcell* and *Snudda* focused on the effect on ion channels and receptors. Hence, we have included a limited part of the effects that neuromodulators have on network activity (Nadim and Bucher, 2014). Many of the targets of neuromodulators are not incorporated into multicompartmental models, including transcription factors and other subcellular processes. Simulations of such effects would require other types of models and simulators. Although, a possible future development would be to include synaptic plasticity within the large scale simulations. Currently, *Snudda* includes receptor models of glutamate (NMDA/AMPA) and GABA receptors with short-term plasticity. In several brain areas, neuromodulators can regulate the long-term potentiation (LTP) and long-term depression (LTD) (Huang et al., 2012; Xu et al., 2018; Perrin and Venance, 2019). Hence, synaptic plasticity could be coupled to neuromodulation levels during the simulation and simulate the effect of LTP vs. LTD, although currently this is not included in *Snudda*.

*Snudda* is a general tool for simulating large scale networks of neurons in any part of the nervous system with NEURON simulator. Our aim was to develop an extension to *Snudda*, which simulates neuromodulation on a large scale and can include any neuromodulator. We then developed *Neuromodcell*, which provides a tool for investigating neuromodulation at single cell level, in addition to the integration into the *Snudda* framework. These tools can provide a link between the single cell experiments and circuit level experiments of neuromodulation, which is currently not possible with the available simulation tools.

## Data Availability Statement

The datasets presented in this study can be found in online repositories. The names of the repository/repositories and accession number(s) can be found below: The code for single neuron optimizations available at github.com/jofrony/Neuromodcell.git and *Snudda.neuro modulation* is available at github.com/Hjorthmedh/Snudda.git.

## Author Contributions

JF designed *Neuromodcell* and generalization of neuromodulation in *Snudda* and wrote the manuscript with some input from the co-authors. JHj, SG, and JH contributed throughout the study by discussing the results. All authors contributed to the article and approved the submitted version.

## Conflict of Interest

The authors declare that the research was conducted in the absence of any commercial or financial relationships that could be construed as a potential conflict of interest.

## Publisher’s Note

All claims expressed in this article are solely those of the authors and do not necessarily represent those of their affiliated organizations, or those of the publisher, the editors and the reviewers. Any product that may be evaluated in this article, or claim that may be made by its manufacturer, is not guaranteed or endorsed by the publisher.

## Footnotes

1
github.com/jofrony/Neuromodcell

2
github.com/neuronsimulator/nrn

3
github.com/Hjorthmedh/Snudda

4
github.com/jofrony/Neuromodulation-software

5
github.com/NeuralEnsemble/elephant
