# Supplementary material for: Dopaminergic and Cholinergic Modulation of Large Scale Networks in silico Using Snudda
Source: Front Neural Circuits. 2021 Oct 21;15:748989. doi: 10.3389/fncir.2021.748989 (PMC8568057; doi:10.3389/fncir.2021.748989)
Supplement: Supplementary file 1 [file Data_Sheet_1.docx]

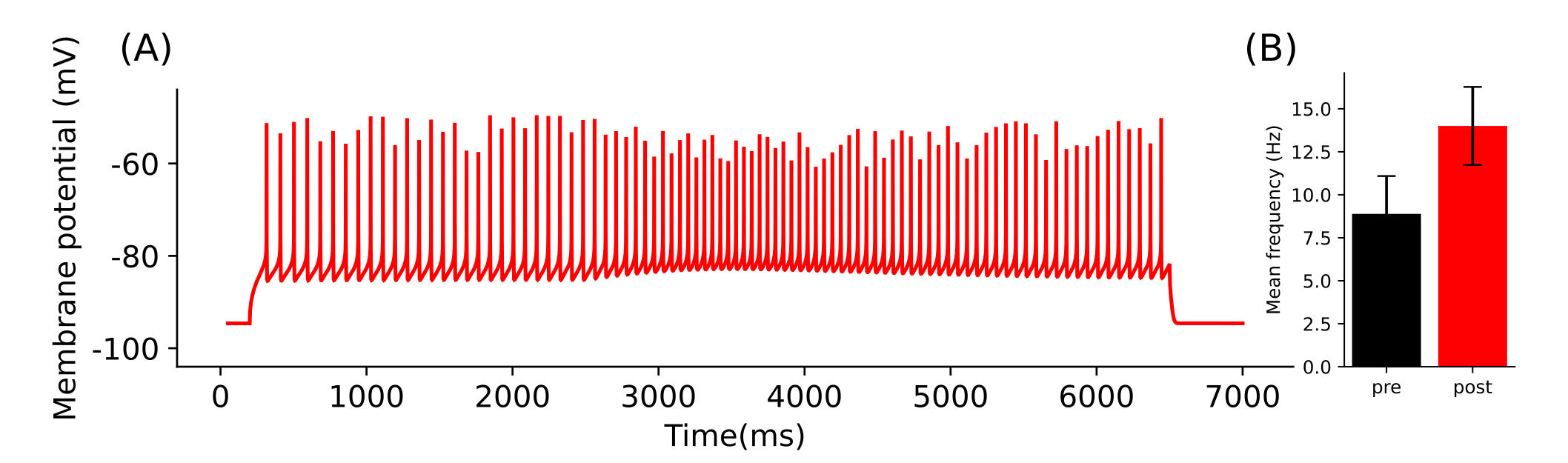
Figure S1: Simulation of dopaminergic modulation reproducing the results from Lahiri and Bevan, (2020). (A) An example trace of a dSPN with step current to induce spiking, as in Lahiri and Bevan, (2020) Figure 1. Dopaminergic modulation at 2500 ms increases the firing frequency of the dSPN. (B) The frequency of pre (before induction of DA modulation) and post (after DA modulation) of 3 dSPN models with mean and standard deviation.


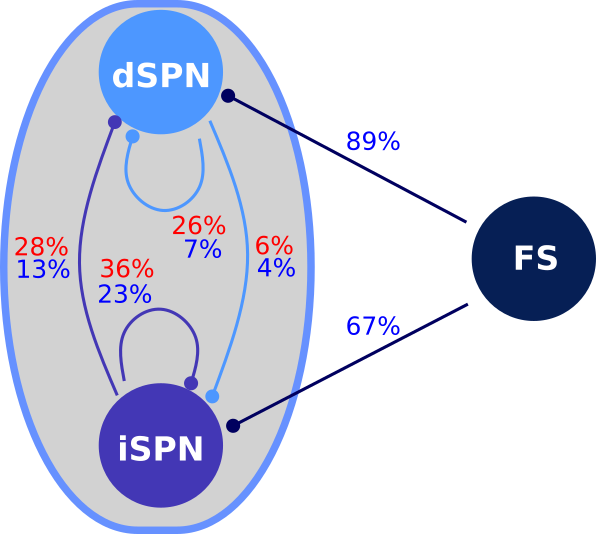


Figure S2: Connectivity diagram of the striatal microcircuit consisting of direct and indirect striatal projection neuron (dSPN and iSPN) and fast-spiking interneuron (FS). The connections are GABAergic and the red and blue connection probabilities between cell pairs below 50 and 100 microns respectively. (Adapted from Hjorth et al. 2020)


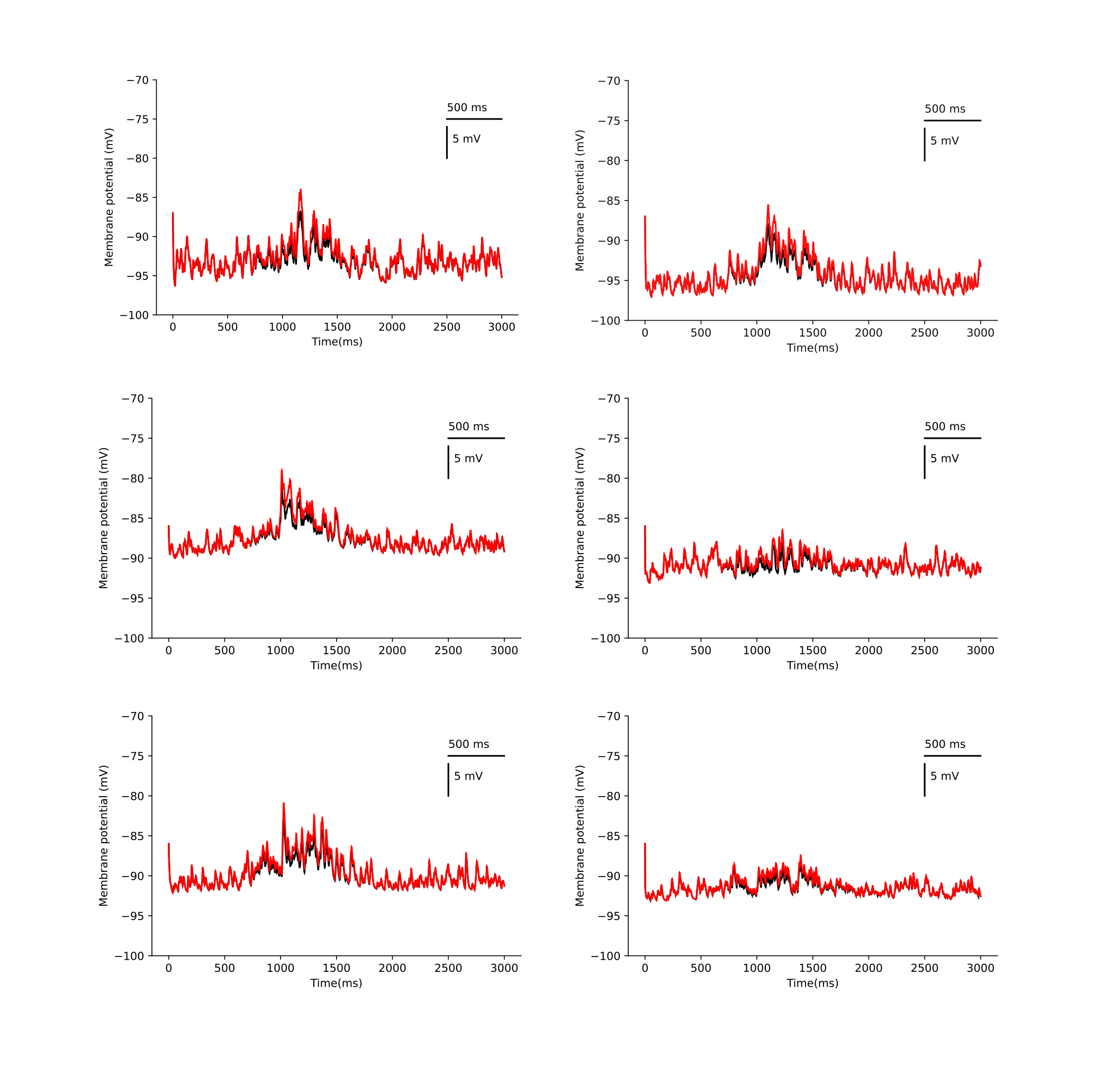
Figure S3 : Dopaminergic modulation of external excitatory input to dSPNs. A simulated cortical activation at 1 s (lasting 500 ms). In red, dopaminergic modulation and in black the control. Dopaminergic modulation of receptor models, which is achieved by increasing the conductance of the glutamate receptor model – tmglut . In control, the cortical stimulation without dopamine modulation. Following dopamine modulation, the amplitude of the synaptic input increases.


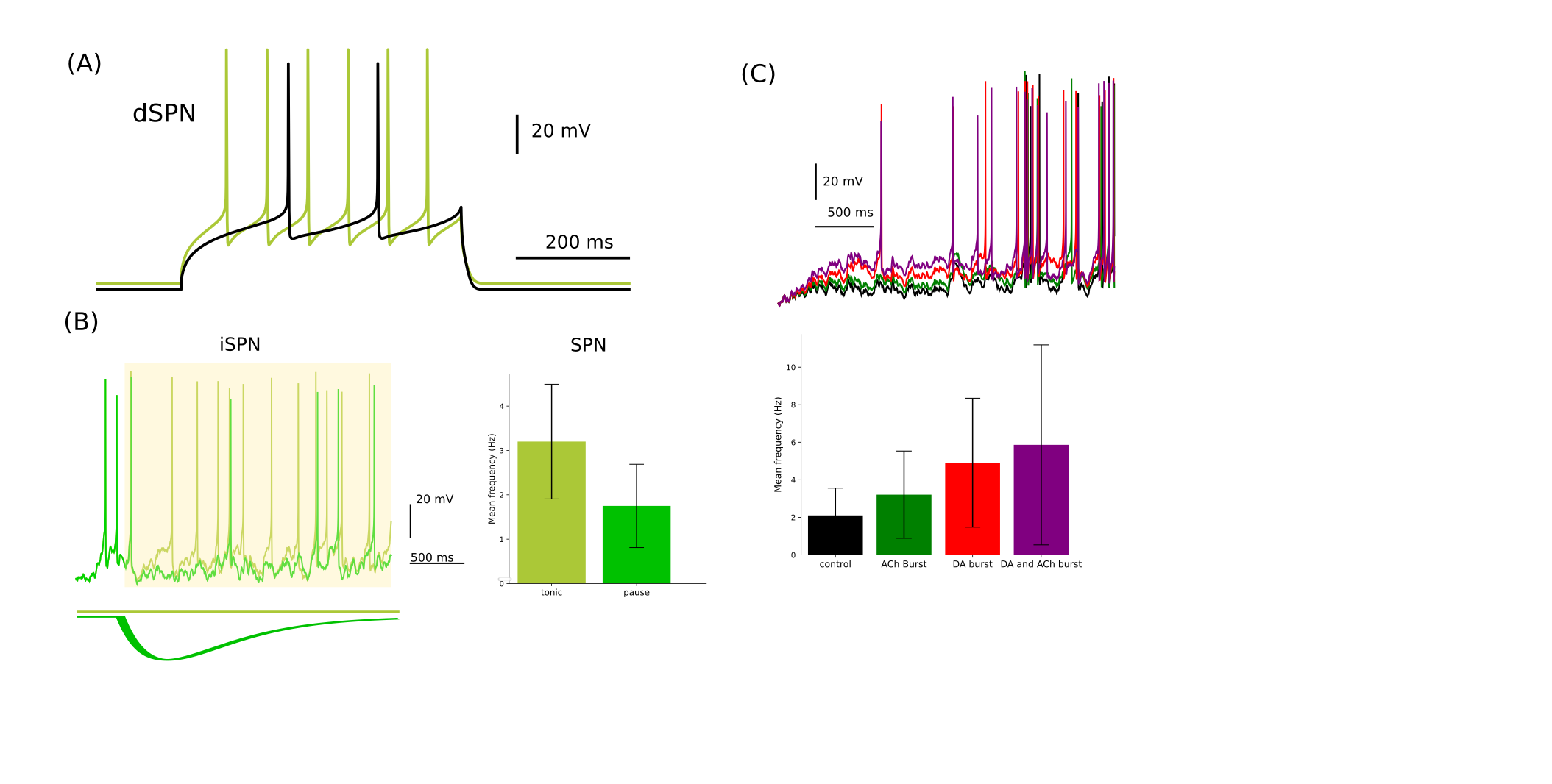


Figure S4 : Muscarinic and dopaminergic modulation of SPNs. (A) Muscarinic modulation of dSPNs following optimization using *Neuromodcell* in black (control) and modulation in green. (B) Simulation of an experiment from Zucca et al. (2018). A tonic level of acetylcholine (ACh) is simulated as control and a pause response in the level of ACh is simulated to replicate the optogenetic inhibition of cholinergic interneurons in Zucca et al. (2018). In the right panel, the change in activity in the pause simulation is seen as a reduction in mean firing frequency (Hz). (C ) A simulation of dSPNs with ACh, DA and DA and ACh burst compared to a control without neuromodulation, with cortical and thalamic background activity. Both ACh and DA burst produce an increase in the mean firing frequency and produce an additive effect in the DA and ACh burst simulation, which is consistent with previous simulations in Lindroos et al. (2020).

| **dSPN** | **iSPN** | **FS** |
| --- | --- | --- |
| **Dopamine (D1 receptor)**   - naf - kas - kaf - kir - cal12 - cal13 - cal13 - can   **Muscarinic (M1 and M4 receptors)**   - kaf - kir - cal12 - cal13 - can - Im | **Dopamine (D2 receptor)**   - naf - kas - kaf - kir - cal12 - cal13 - cal13 - can - car   **Muscarinic (M1)**   - kir - cal12 - cal13 - can - Im | **Dopamine (D1 receptor)**   - kir - kas - kaf - naf |

Table S1: List of ion channels modulated by dopamine and acetycholine (muscarinic) for direct and indirect striatal projection neuron (dSPN and iSPN) and fast-spiking interneuron (FS). Taken from previous publication Lindroos et al. 2018, Hjorth et al. 2020 and Lindroos et al 2020. M4 receptor (muscarinic acetylcholine receptor M4); M1 receptor (muscarinic acetylcholine receptor M1); D1 (Dopamine receptor D1); D2 (Dopamine receptor D1)
